# *Blumea balsamifera* Oil for the Acceleration of Healing of Burn Injuries

**DOI:** 10.3390/molecules200917166

**Published:** 2015-09-17

**Authors:** Zuo-Wang Fan, Yu-Xin Pang, Kai Wang, Fu-Lai Yu, Dan Wang, Quan Yang, Qing-Song Ma, Xiao-Ting Li, Jin Zou, Wen-Qing Zhang, Li-Fen Wu

**Affiliations:** 1 Tropical Crops Genetic Resources Institute, Chinese Academy of Tropical Agricultural Sciences, Danzhou 571737, China; E-Mails: fujianfanzuowang@126.com (Z.-W.F.); jimojijie29@163.com (K.W.); fulai.yu@163.com (F.-L.Y.); maqing_song@126.com (Q.-S.M.); lxt2015cool@126.com (X.-T.L.); zj602395791@126.com (J.Z.); 13602428576@163.com (W.-Q.Z.); w499494265n@126.com (L.-F.W.); 2 School of Traditional Chinese Medicine, Guangdong Pharmaceutical University, Guangzhou 510006, China; E-Mail: yangquan7208@vip.163.com; 3 Key Laboratory of Crop Gene Resources and Germplasm Enhancement in Southern China, Danzhou 571737, China; 4 Hainan Provincial Engineering Research Center for *Blumea balsamifera*, Danzhou 571737, China

**Keywords:** *Blumea balsamifera* (L.) oil (BBO), deep second-degree burn, inflammatory factor, cytokine, growth factor

## Abstract

*Blumea balsamifera* oil (BBO) is a main extract obtained from *Blumea balsamifera* (L.) DC (Ainaxiang) leaves, which are widely used as a traditional medicine by the Miao and Li Nations to promote skin trauma or burn injury healing. This study was initiated to investigate the healing efficacy in deep second-degree burn model in rats. The rats were treated by BBO for 21 consecutive days. The rate of healing, scabs dropped time and re-epithelialization time were observed every three days for 21 days after burn injury. The samples were collected from different treated rats by sacrificing the animals on the 1st, 2nd, 5th, 9th, 14th, and 21st day post-burn creation. Then, the water content of burn tissue was measured. Plasma interleukin-1 (IL-1) and tumor necrosis factor-alpha (TNF-α) levels were evaluated, and the tissue expressions of basic fibroblast growth factor (bFGF), vascular endothelial growth factor (VEGF), and transforming growth factor-beta (TGF-β) were determined along with skin histopathology. The results showed that the water content of tissue was significantly reduced, the scabs dropped time shortened, and healing accelerated after treatment with BBO in the burn injury rats. Furthermore, the expressions of growth factors were significantly increased in the tissue; however, the levels of inflammatory factors on plasma decreased. This study confirms the efficacy of BBO consumption on burn injuries.

## 1. Introduction

Burn injuries, caused by heat, light, electricity, radiation and chemicals, are one of the most common and devastating forms of trauma, particularly partial- and full-thickness burns [1]. According to the report of Institute of Burn Third Military Medical University, about 5000–10,000 in one million people suffered from burn in China every year [2]. Depending on the severity, burn injuries are classified into three degrees, whereby the second degree is divided into shade and deep degrees [3]. Burn-wound could result in the increase of free radical-mediated damage and reactive oxygen species. It can delay granulation tissue formation, reduce angiogenesis and decrease collagen reorganization. Furthermore, due to its significantly prolonged period of rehabilitation, the healing of burn injuries is more difficult than that of ordinary wounds and is also associated with higher economic costs [4,5].

Current methods to treat burn injuries include antibiotics, antiphlogistics, and silver salt, which possesses major drawbacks and unwanted side effects [6,7,8,9,10]. Nowadays, the alternative and complementary medicines, such as traditional Chinese medicine and aromatherapy, are being used, because they are moderately beneficial to effective with little toxicity and are less expensive than synthetic drugs. Many plants and plant-derived products have been found to possess potent wound-healing or burn-wound-healing activity [11,12,13,14].

*Blumea balsamifera* (L.) DC., (*B. balsamifera*) an important medicinal herb the has been used as a traditional ethnic medicine for thousands of years in China, preferentially grows in the wild throughout Southeast Asia, such as Hainan, Guizhou, Yunnan, Guangdong, and Taiwan provinces [15,16,17]. In some ancient Chinese minorities such as Li, Miao and Zhuang, the whole plant or its leaves were used to treat eczema, dermatitis, beriberi, lumbago, menorrhagia, rheumatism, skin injury, and as an insecticide [18,19]. Previous studies suggested that leaf extracts display antibacterial [20], plasmin-inhibitory [21], free radical-scavenging [22], NO inhibitory [23] and anticancer [24,25] activities. Phytochemical analysis revealed that *B. balsamifera* leaves contained considerable amounts of volatile oil and flavonoids. In addition, the volatile oil analysis by GC-MS showed that its dominant constituents were l-borneol, d-camphor, caryophyllene and ledol [26,27,28]. As an important plant source of l-borneol, *B. balsamifera* was appointed as the only plant source for *Aipian* by Pharmacopoeia of the People’s Republic of China (2010) [29,30]. BBO was produced from industrialized *Aipian*, and used to treat burn-wound healing by the local ethnic minorities in Guizhou, in the southwest of China [19,31].

However, several questions still remain unanswered: Is the essential oil from *B. balsamifera* really effective for burn-wound healing? How does it generate the salutary effects? What are the mechanisms of action? With these queries in mind, a series of experiments were designed to investigate the effect of BBO on burn injuries.

## 2. Results and Discussion

### 2.1. Effect of Different Treated Groups of BBO on General Observation in Rats

**Figure 1:**
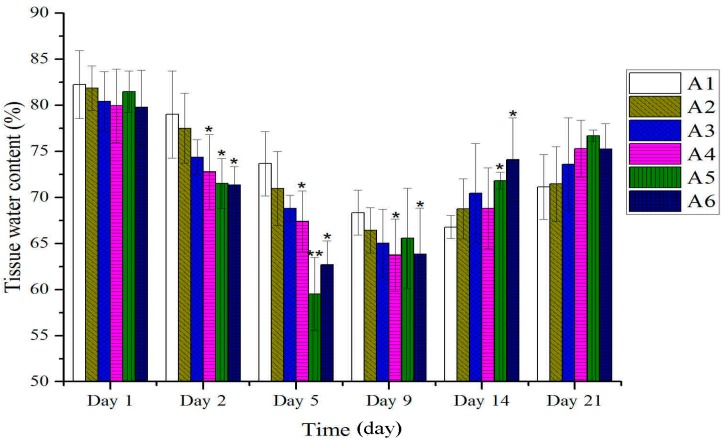
Effect of different concentration or fractions of BBO on the tissue water content in rats. Values are expressed as mean ± S.D. (*n* = 4 animals). *: *p* < 0.05 *vs.* control; **: *p* < 0.01 *vs.* control.

**Figure 2:**
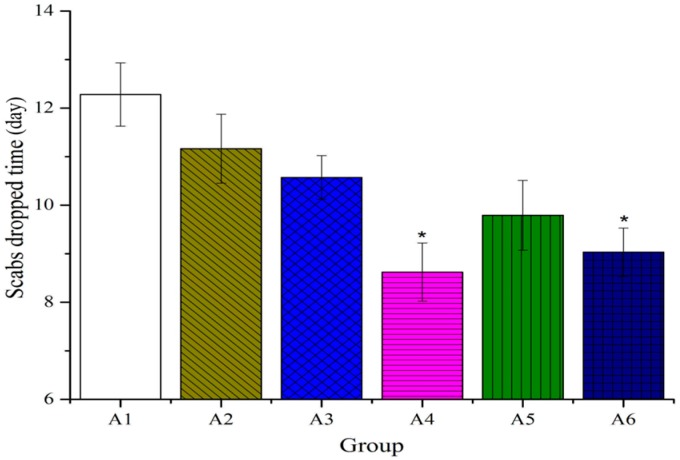
Effect of different concentration or fractions of BBO on the scabs dropped time in rats. Values are expressed as mean ± S.D. (*n* = 4 animals). *: *p* < 0.05 *vs.* control.

**Figure 3:**
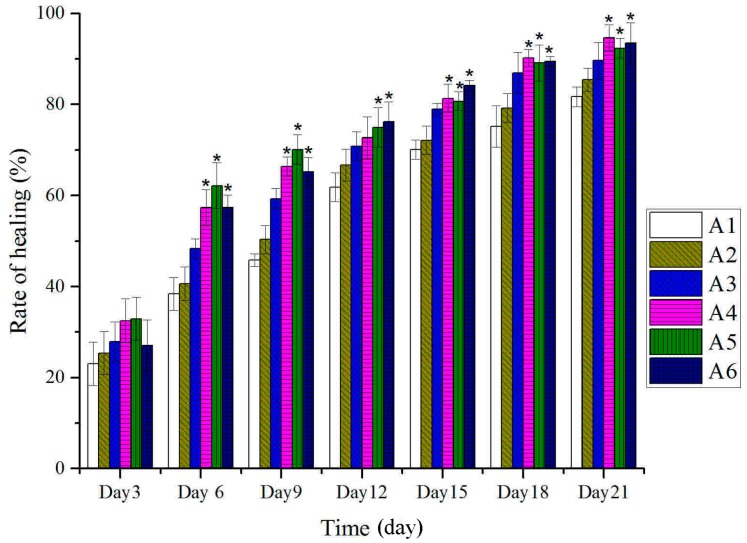
Effect of different concentration or fractions of BBO on the rate of wound healing in rats. Values are expressed as mean ± S.D. (*n* = 4 animals). *: *p <* 0.05 *vs.* control.

As shown in Figure 1, no remarkable differences were observed between treated and untreated groups in the tissue water content on the first day after burn. However, on the second day after burn, the percentage of water content significantly decreased compared to the control group. The scabs dropped time was 12.28 days (range, 11–13 days), control group; 11.16 days (range, 10–12 days), 80% ethanol (vehicle) group; 10.57 days (range 9–11 days), burn ointment (standard group); 9.15 days (range 8–10 days), BBO groups (Figure 2). On the fourth day after burn, the rate of wound healing treated with BBO was significantly greater than the control group. The burn wound was almost completely healed (>90%) on the 21st day (Figure 3), the re-epithelialization time correspondingly shortened.

### 2.2. Effect of Different Treated Groups of BBO on Plasma IL-1 and TNF-α Level in Rats

**Figure 4:**
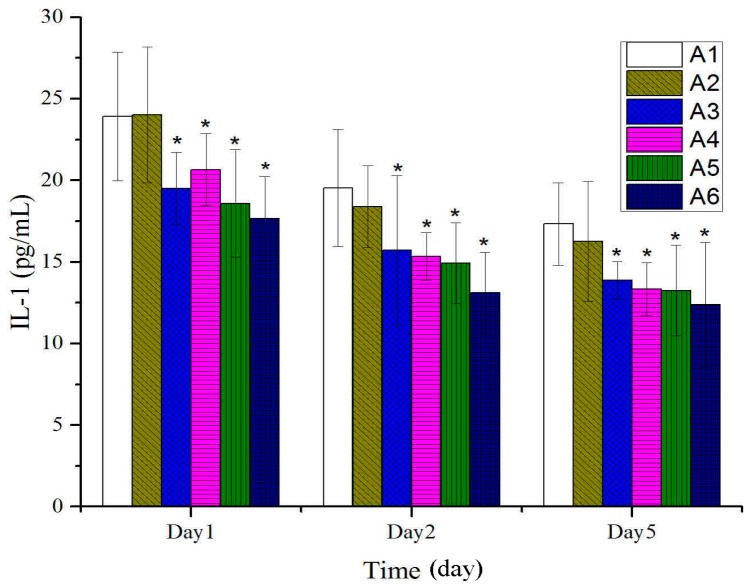
Effect of different concentration or fractions of BBO on plasma IL-1 in rats. Values are expressed as mean ± S.D. (*n* = 4 animals). *: *p* < 0.05 *vs.* control.

**Figure 5:**
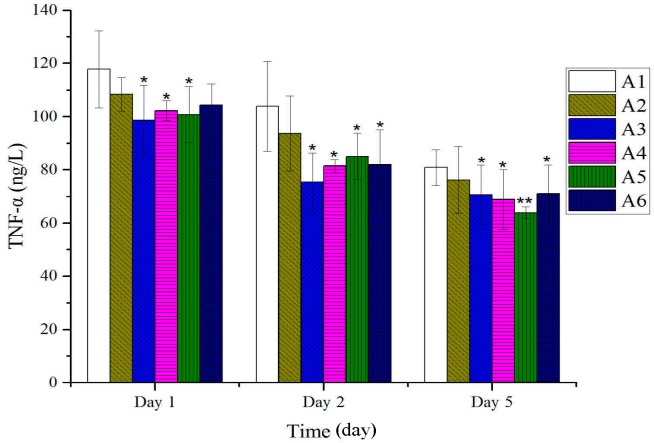
Effect of different concentrations or fractions of BBO on plasma TNF-*α* in rats. Values are expressed as mean ± S.D. (*n* = 4 animals). *: *p* < 0.05 *vs.* control; **: *p* < 0.01 *vs.* control.

As shown in Figure 4, a prominent decrease in IL-1 was observed with each BBO treatment five days after burn. As shown in Figure 5, the levels of TNF-α on plasma rapidly declined after being treated with different concentration or fractions of BBO five days after burn. No remarkable differences were observed between treated and untreated groups on the fifth day after burn.

### 2.3. Expressions of bFGF in Tissues after Treatment with BBO in Rats

**Figure 6:**
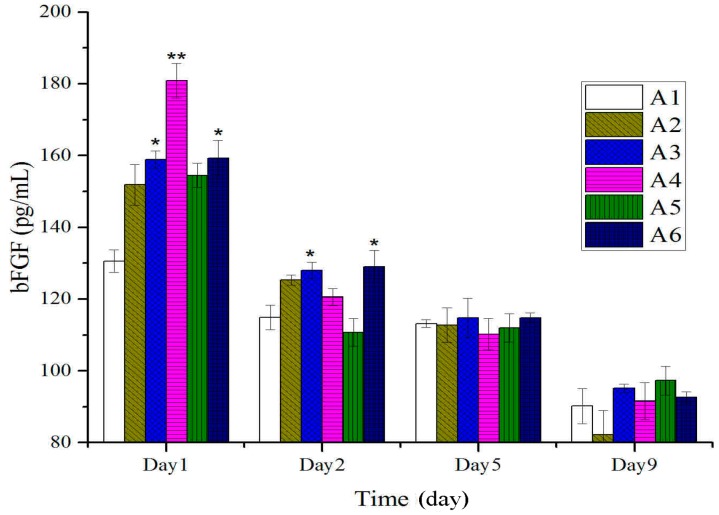
Expressions of bFGF in tissues after treatment with BBO in rats. Values are expressed as mean ± S.D. (*n* = 4 animals). *: *p* < 0.05 *vs.* control; **: *p* < 0.01 *vs.* control.

As shown in Figure 6, at the early stage after burn (first day), expressions of bFGF of treated groups with different concentration of BBO were significantly higher than those of the control group and vehicle group. With prolonging of the treatment time, there were no significant shifts in the bFGF levels at different groups.

### 2.4. Expressions of TGF-β and VEGF in Tissues after Treatment with BBO in Rats

**Figure 7:**
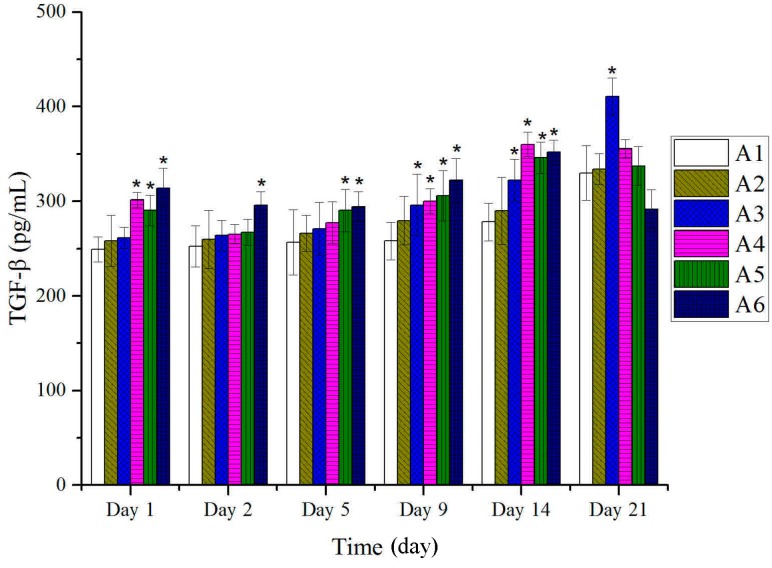
Expressions of TGF-β in tissues after treatment with BBO in rats. Values are expressed as mean ± S.D. (*n* = 4 animals). *: *p* < 0.05 *vs.* control.

As shown in Figure 7, at the first period of burn (first day), the expressions of TGF-β of treated groups with BBO were significantly different compared to those of the control and vehicle groups. However, the levels of TGF-β reduced before the burn-skin dropped. With the beginning of re-epithelialization, the expressions of TGF-β were significantly higher than those of control and vehicle groups, and reached the peak at 14th day.

**Figure 8:**
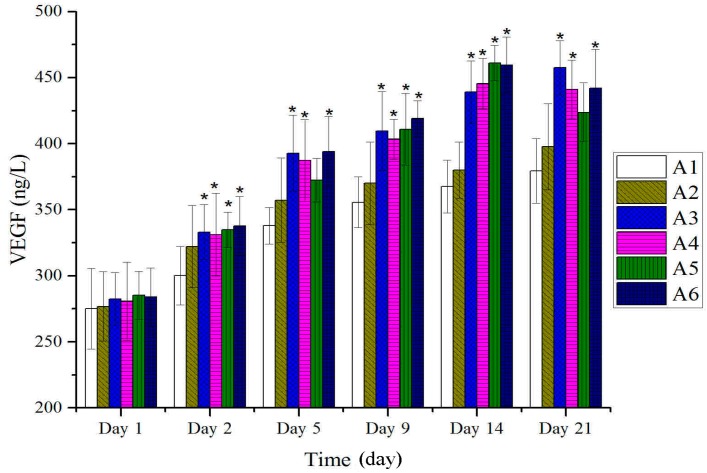
Expressions of VEGF in tissues after treatment with BBO in rats. Values are expressed as mean ± S.D. (*n* = 4 animals). *: *p* < 0.05 *vs.* control.

As shown in Figure 8, expressions of VEGF of all the treated groups with BBO showed no significantly difference compared to those of the control group and vehicle group at the early stage after burn injury. A significant increase in expressions of VEGF was observed at the start of re-epithelialization, and until the wound healing (*p* < 0.05). Thus, the expression of VEGF increased significantly to enhance the burn-wound healing.

### 2.5. Histological Analysis

The burned tissues were measured by histological analysis to confirm the burn degree. The pathological sections showed that there were neither dermis nor epidermis; deep second-degree burn was obtained [32,33].

**Figure 9:**
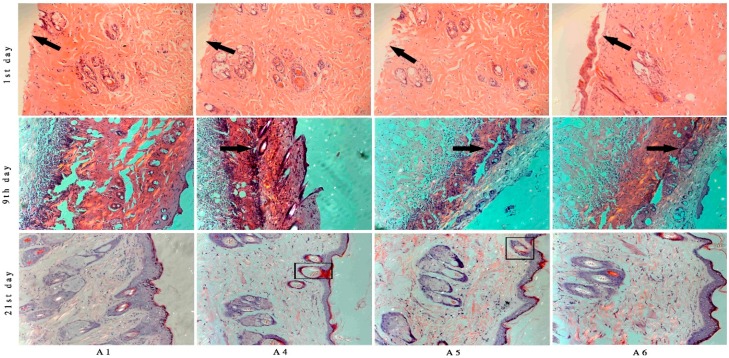
Histopathology of burned tissues samples after treatment with different concentration of BBO at Days 1, 9 and 21 (stained with Hematoxylin-Eosin). On the first day after burn, the pathological sections showed that there were neither dermis nor epidermis (arrows at first day). At the ninth day, the scabs were dropped, and some new epithelial tissues were observed (arrows at ninth day). At the 21st day, the wound was covered by healthy epithelial tissue and the new epidermis, and generated the sebaceous and sweat gland (blocks at 21st day).

The histological sections from BBO treated rats showed more collagen fibers and fibroblasts cells on ninth day after burn injury compared with the control group (Figure 9). On the 21st day after burn injury, the wounded tissue treated with different concentration of BBO was almost (>90%) covered with healthy epithelial tissue and the new epidermis. The rats under different concentrations of BBO treatments showed complete epithelialization of the burn injury area, and the sebaceous gland in the section was observed (Figure 9).

### 2.6. Discussion

Burn is a common injury in daily clinical practice. Despite the advanced clinical care and reduced mortality of scalded patients [30,31], some problems still existed such as burn wound infection, delayed wound healing and hypertrophic scarring [34]. Patients were also faced with high economic costs as well. Alternative or complementary medicines, such as traditional Chinese medicine or ethnic drugs, have been used to improve this situation [35].

In this study on rats, different concentrations of BBO have been used and showed that these could significantly accelerate the burn-wound healing. The anti-inflammatory was a main response in the body after burn injuries. The tissue water content was a key index in anti-inflammatory, and its decrease displayed inflammatory was weakened. The percentage of tissue water content was detected and significantly decreased compared to the control group on the second day after burn, especially the 50% BBO group on the fifth day after burn (*p* < 0.01). The levels of inflammatory factors such as IL-1 and TNF-α on plasma were analyzed at the early stage of burn (Figure 4 and Figure 5). Previous studies suggested that IL-1 and TNF-*α* accelerate the anti-inflammatory at low concentration at the early stage of burn, and high concentration could cause systemic inflammatory response syndrome [36,37,38,39]. These results confirmed significant decrease in the levels of IL-1 and TNF-α of rats after treatment with BBO on plasma (*p* < 0.05, respectively), and especially the 50% BBO was observed at fifth day after burn (*p* < 0.01). In the present study, the treated groups with BBO were found to promote anti-inflammatory activity by reducing the levels of inflammatory factors.

Many types of cytokines and growth factors are responsible for inflammation, re-epithelialization, wound contraction, the formation of granulation tissue and neovascularization during the burn-wound healing process [40]. TGF-β plays a key role through regulating different cellular functions, and enhancing granulation tissue formation and collagen formation [41]. In addition, TGF-β has also been reported to be able to promote wound contraction through its direct induction of alpha smooth muscle act in expression in fibroblasts [42,43]. At the same time, VEGF as an important growth factor in pathological situations, such as tissue repair, involves neovascularization and increased vascular permeability [44,45]. In addition, VEGF also mediates vascular hyper permeability and promotes the secretion of both active growth factors and cytokines necessary for wound repair [46,47,48,49]. These results suggested that the expressions of TGF-β and VEGF followed the beginning of re-epithelialization and significantly higher in the BBO treated groups, and reached the peak on the 14th day (Figure 7 and Figure 8).

A previous study by Werner S showed that bFGF was confirmed as a trigger in the wound repair to enhance the expression of TGF-β and VEGF [44]. Further, it was also an important growth factor in the early phase of burn injury, to improve the quality of skin grafting in burn patients. In this study, the rats treated with BBO significantly enhanced the expression of bFGF at the early stage of burn injury, stimulating the formation of collagenous fiber and the expressions of TGF-β and VEGF (Figure 6). It is important to mention that BBO could accelerate the burn-wound healing by influencing the expression of growth factors and cytokines.

This investigation suggested that three different concentrations of BBO were able to promote burn-wound healing, and the rate of healing in the treated group with 10% BBO was faster than in 50% BBO group (Figure 3). It may be influenced by ethanol (80%), which was a good germicide and vehicle. However, the results of vehicle group showed that ethanol (80%) could not significantly accelerate the wound healing. It revealed that germicide as an auxiliary with could produce better therapeutic activity only with the help of BBO. Compared with the quality of skin that recovered from burn injury, the skin treated by 100% and 50% BBO groups was superior to that of other treated groups. The new skin has been covered with healthy epithelial tissue and the new epidermis, and generated the sebaceous and sweat gland with little visible scars (Figure 9). The skin thickness of new epidermis with BBO groups was significantly thinner compared to the control group (Figure 9). It may be related to the secretion of cytokines and growth factors and this needs further investigation [50,51,52].

Though some papers published by other authors reported that the BBO excited different components caused by location and extraction procedure, the main composition was uniform, for example l-borneol, d-camphor and caryophyllene, which makes sure that BBO is uniformly efficacious as an effective burn-healing agent [26,27,28,53]. Previous investigation showed that l-borneol and d-camphor were the main constituents in BBO [28]. l-borneol has been reported to be able to promote permeability and is anti-inflammatory, inhibiting the expressions of TNF-α, IL-1 and IL-6 [54,55,56,57,58]. However, the contact between l-borneol and growth factors is not reported. It may also be the main effect ingredient that could accelerate anti-inflammatory effects at the early stage of burn injury healing. Possibly, the healing efficacy of BBO may probably be due to the synergistic action of its multiple phytoconstituents, and further phytochemical studies are required to isolate the active compound(s) responsible for adding these pharmacological activities.

## 3. Experimental Section

### 3.1. Materials

*B. balsamifera* leaves was collected in Luodian (Guizhou, China) and BBO was provided by Guizhou AiYuan Ecological Medicine Development Ltd. (Guizhou, China). The oil yield was about 0.01% of fresh *B. balsamifera* leaves and extracted by steam distillation. The samples were stored at 4 °C until use and the composition were analyzed in previous investigation [28].

### 3.2. Experimental Animals

Healthy male and female Sprague-Dawley rats were obtained from Changsha Tianqin Biotech Ltd. (Changsha, China). The rats, weighing between 220 and 250 g, were housed in polypropylene cage and maintained in standard laboratory conditions of temperature (24 ± 2 °C), light-dark cycle 12 h:12 h and humidity (60% ± 5%). They were fed on standard pellet diet and water *ad libitum*. Before administering any treatment, rats were kept off feed for 7 days. After treatment, food was made available *ad libitum*. Then, the animals were sacrificed under anesthesis at the end of the experiment.

### 3.3. Animal Model

Rats were anesthetized with 10% chloral hydrate with a dose of 350 mg kg^−1^ of body, administered by an intra-peritoneal injection. The dorsal fur was removed by 10% sodium sulfide. Briefly, a 2.5 cm in diameter steel rod (25 cm in length) was heated to 100 °C in boiling water for 15 min, then it was applied for 7 s on the removed dorsal skin of the rat and created two full thickness of burn injury. Lactated Ringer’s solution (5 mL of body) was immediately injected into peritoneal cavity to prevent shock [59]. All animals were randomly assigned to 6 groups of 24 each. After 1 h, all the animals were administered treatment (Table 1) and applied once daily for 21 days.

**Table 1:** Animal groups (*n* = 24/group), treatments, doses and route of administration of different concentration or fractions of BBO TDD.

Group No.	Treatments	Doses (mg/kg)
A1	Control	0
A2	80% ethanol (vehicle)	1500
A3	Moist exposed burn ointment (standard)	1500
A4	BBO (100%)	1500
A5	BBO (50%)	1500
A6	BBO (10%)	1500

### 3.4. General Observation

The rate of healing, scabs dropped time, granulation and re-epithelialization time were observed every three days for 21 days after burn. The full-thickness of burn area (about 100 mg) was removed from different treated rats by sacrificing the animals on the 1st, 2nd, 5th, 9th, 14th, and 21st day post-burn creation, and weight was measured after bake for 24 h at 80 °C to obtain the tissue water content. The rate of healing was assessed by digitized planimetry.

### 3.5. Sample Collection

Blood samples (about 3 mL) were collected from rats by vein puncture in the mandibular region under anesthesia on the 1st, 2nd, 5th, 9th, 14th, 21st day post-burn creation, and centrifuged at 4000 rpm for 10 min at 15 °C for plasma. The blood samples were stored at −80 °C in aliquots until analysis.

The full thickness of burn area was removed from different treated rats by sacrificing the animals on the 1st, 2nd, 5th, 9th, 14th, and 21st day post-burn creation, one aliquot (about 100 mg) was processed by standard histological techniques, and others samples were stored at −80 °C in aliquots until analysis.

### 3.6. Clinical Chemistry

Plasma levels of TNF-*α*, IL-1 were analyzed with the TNF-*α* adduct ELISA Kit (Nanjing Jiancheng Bioengineering Institute, Nanjing, China) and the IL-1 Assay kit (Nanjing Jiancheng Bioengineering Institute, Nanjing, China). The frozen full-thickness samples were processed by standard ELISA techniques. The samples were subsequently centrifuged and the supernatant isolated for analysis. The enzyme-linked immunoassay (Nanjing Jiancheng Bioengineering Institute, Nanjing, China) was performed according to the manufacturer’s instructions. The tissue supernatant was analyzed with antibody magnetic beads in duplicate. Three different cytokines and growth factors were assessed, namely, TGF-β, VEGF, and bFGF.

### 3.7. Histopathologic Evaluation

The full-thickness tissue was processed routinely and embedded in paraffin blocks. Tissue sections were prepared (6 μm) and stained with hematoxylin and eosin (H & E). The slides were assessed using a light microscope (200×).

### 3.8. Statistical Analysis

The results were expressed as mean ± standard deviation (SD). Data were analyzed by one-way ANOVA followed by Duncan’s multiple range test using Statistical Package for Social Sciences 18.0 (SPSS, Chicago, IL, USA). Values were considered statistically significant when *p*-value was less than 0.05.

## 4. Conclusions

The study showed that the BBO facilitated wound healing in experimental animal model by influencing the anti-inflammatory response, and the expressions of cytokines and growth factors in burn injury repair process. BBO could upgrade the quality of skin that recovered from burn injuries. The biological data obtained in the present study should be useful for the further development of new burn-treating drugs. However, there is a need for further studies in order to analyze the active ingredients in the BBO and to completely elucidate the mechanisms of actions of these active ingredients.
